# Supplementary figures and images for: LINE-1 retrotransposition in a mouse TDP-43 model of neurodegeneration marks motor cortex neurons for cell-intrinsic and cell non-autonomous programmed cell death
Source: PLoS Genet. 2025 Dec 29;21(12):e1012007. doi: 10.1371/journal.pgen.1012007 (PMC12758826; doi:10.1371/journal.pgen.1012007)

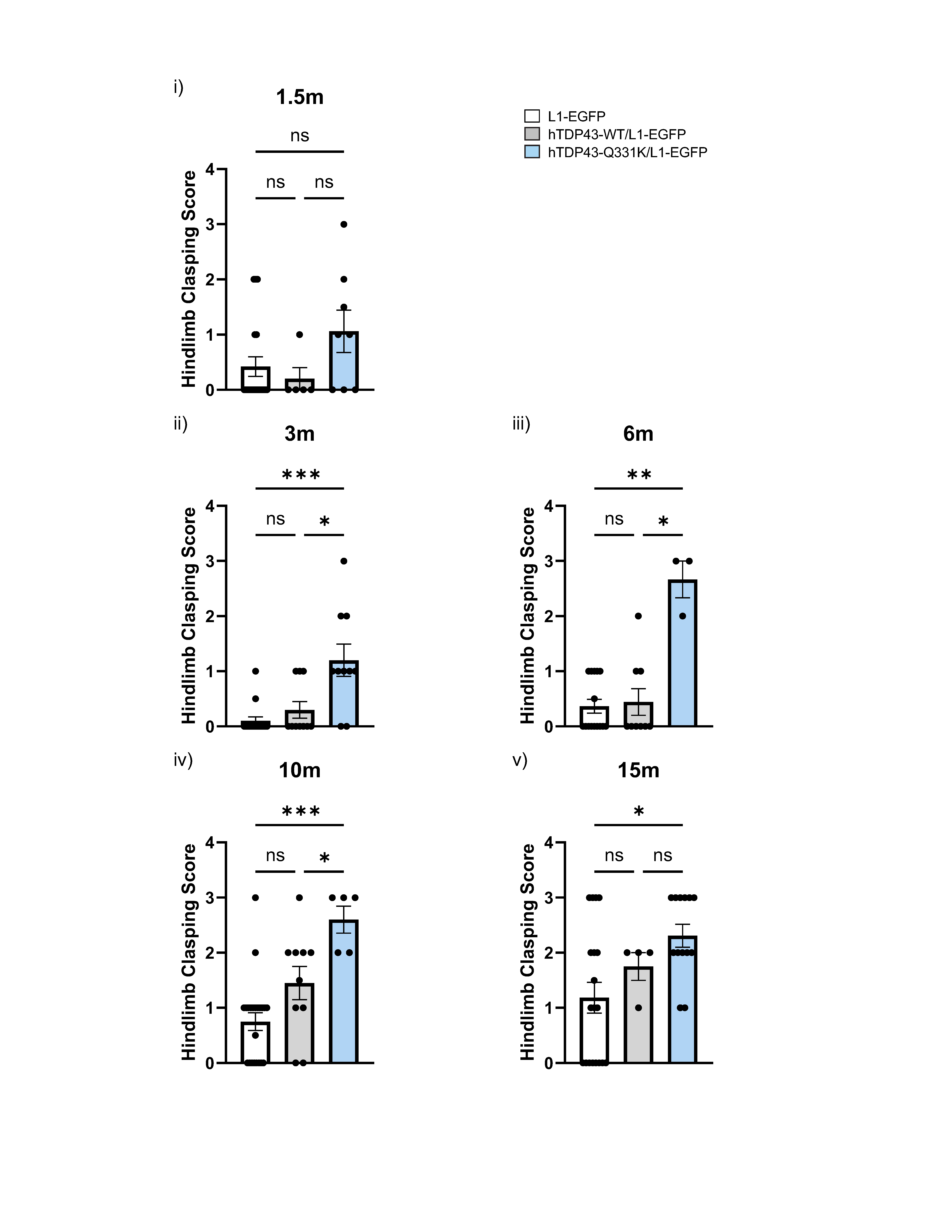

Supplement: S1 Fig — (i-v) Hindlimb Clasping at 1.5, 3, 6, 10, and 15 months. Kruskal- Wallis and Dunn’s multiple comparisons test was used with * p ≤ 0.05, ** p < 0.01, *** p < 0.001, **** p < 0.0001. n = 4–9 animals for each group were used. (ii) p = 0.0008 for hTDP-43-Q331K/L1-EGFP vs L1-EGFP, and p = 0.0316 for hTDP-43-Q331K/L1-EGFP vs hTDP-43-WT Tg/L1-EGFP (iii) p = 0.0098 for hTDP-43-Q331K/L1-EGFP vs L1-EGFP, and p = 0.0155 for hTDP-43-Q331K/L1-EGFP vs hTDP-43-WT Tg/L1-EGFP (iv) p = 0.0013 for hTDP-43-Q331K/L1-EGFP vs L1-EGFP (v) p = 0.0202 for hTDP-43-Q331K/L1-EGFP vs L1-EGFP. (TIF) [file pgen.1012007.s001.tif]

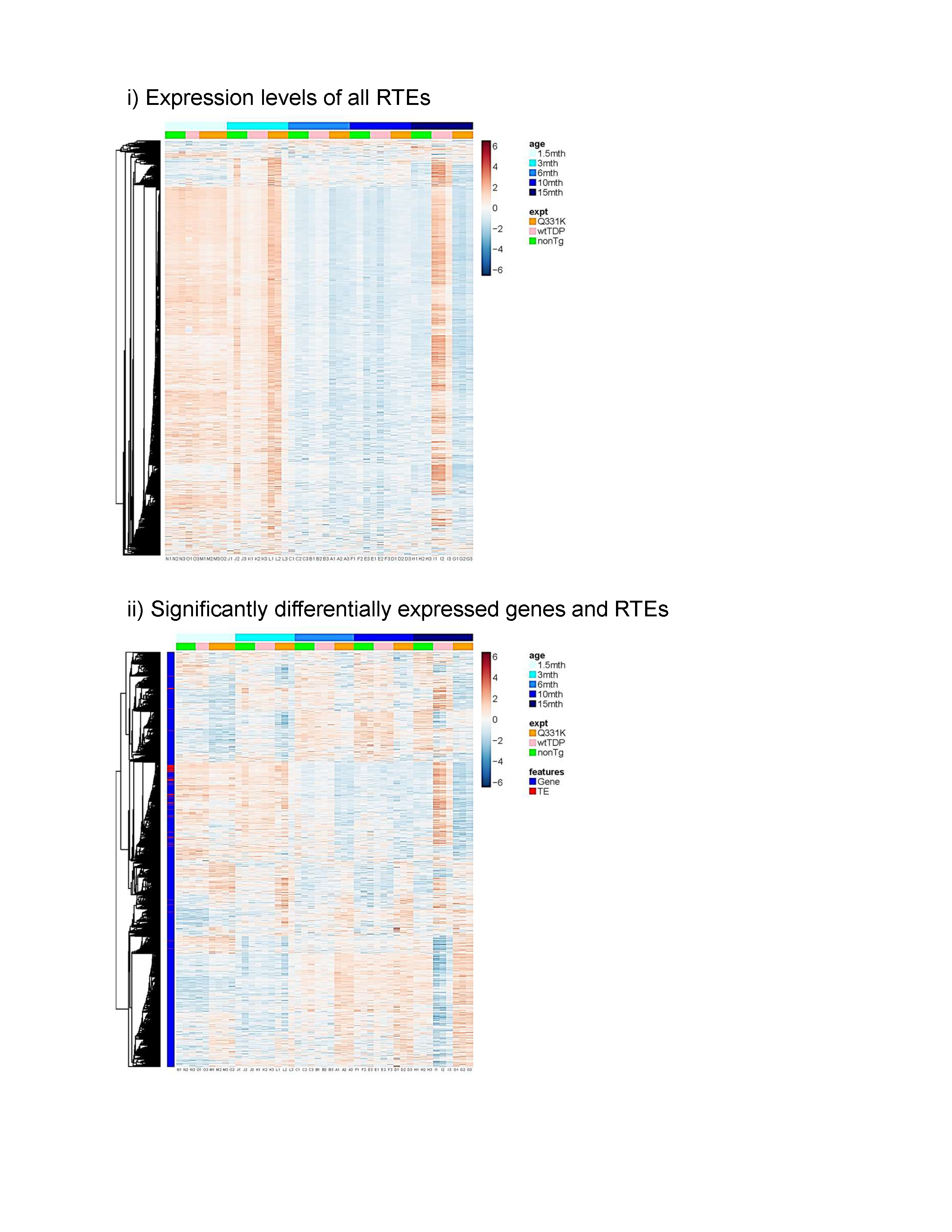

Supplement: S2 Fig — (i) Expression of all RTEs across 1.5, 3, 6, 10, and 15 months in hTDP-43-Q331K Tg and hTDP-43-WT Tg mouse MC. (ii) Significantly differentially expressed genes and RTEs at 1.5, 3, 6, 10, and 15 months in hTDP-43-Q331K Tg and hTDP-43-WT Tg mouse MC. N = 3 mixed-sex, age-matched cohorts were used for all genotypes and age groups except 1.5 month, where n = 2 for nTg and n = 4 for hTDP-43-WT Tg were used. See Methods for details regarding the analysis pipeline, including statistical analyses. (TIF) [file pgen.1012007.s002.tif]

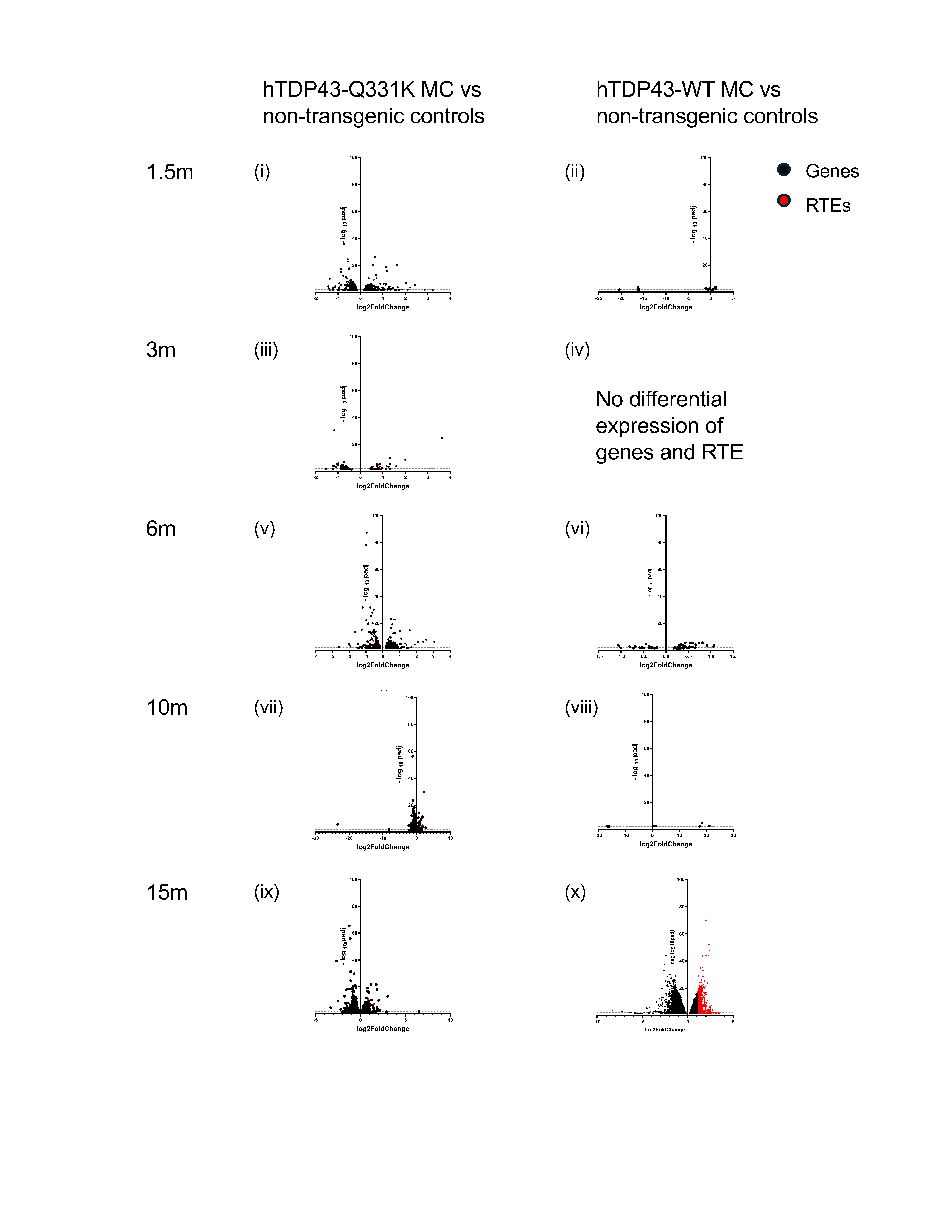

Supplement: S3 Fig — (i–x) Volcano plots showing differential expression of genes and RTE in hTDP-43-Q331K and hTDP-43-WT MC compared to non-transgenic littermates at different time points. The grey dotted line indicates –log10padj value of 2. Legend: black = genes, red = RTE. (TIF) [file pgen.1012007.s003.tif]

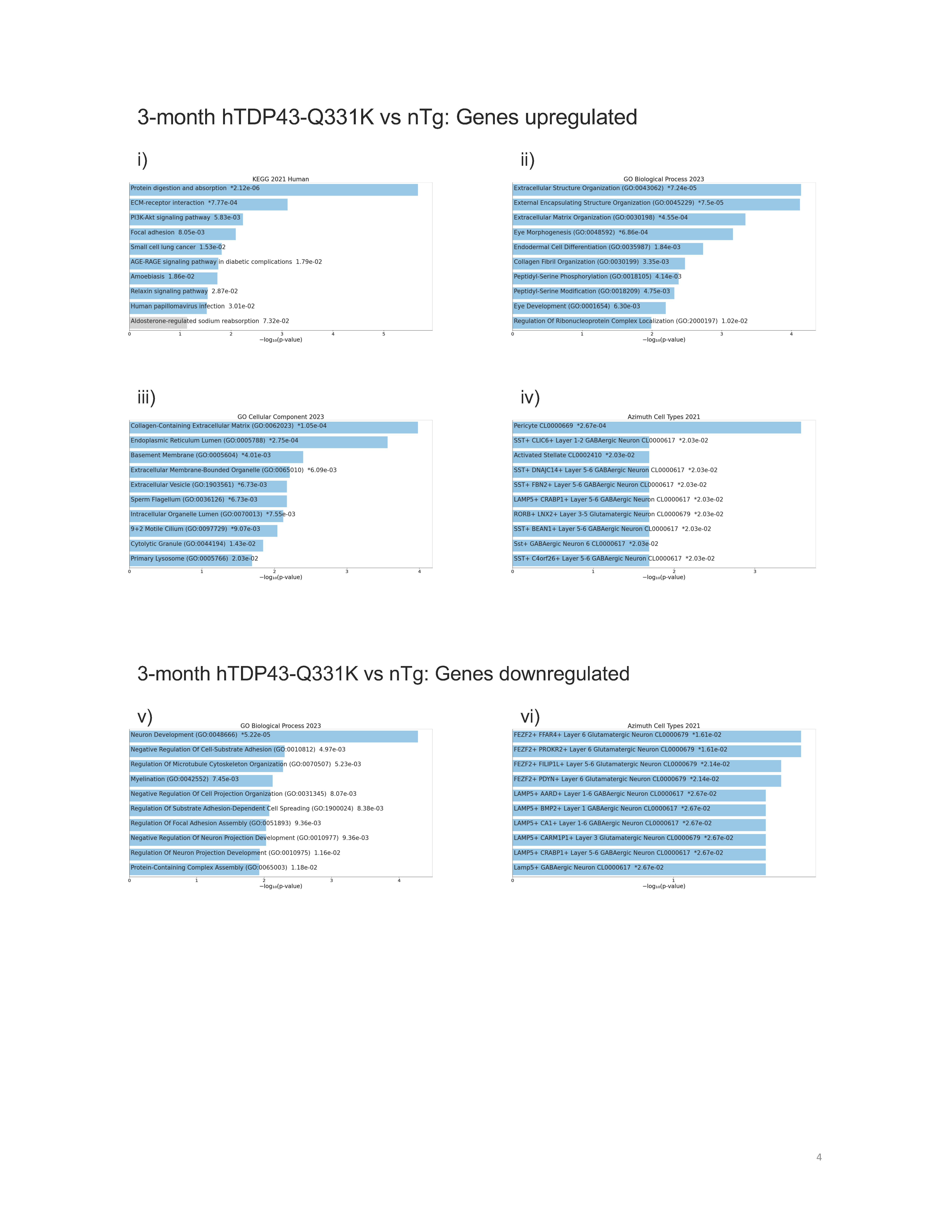

Supplement: S4 Fig — X- axis represents -log10(Padj) values for each component. In the MC of the 3 month-old hTDP-43-Q331K mice, the differentially expressed transcripts showed enrichment for genes involved in extracellular matrix (ECM) organization. By contrast, there was a depletion of genes associated with neuronal development. Significant differential expression was also identified in genes specific to GABAergic and glutamatergic neuron populations, with some genes being upregulated and some being downregulated, in comparison to the Azimuth annotated reference dataset. In contrast to the hTDP-43-Q331K animals, no significant differences were observed in the total RNA from the MC of 3 month-old hTDP-43-WT mice when compared to their non-transgenic littermates. Figures generated on Enrichr [87–89]. (TIF) [file pgen.1012007.s004.tif]

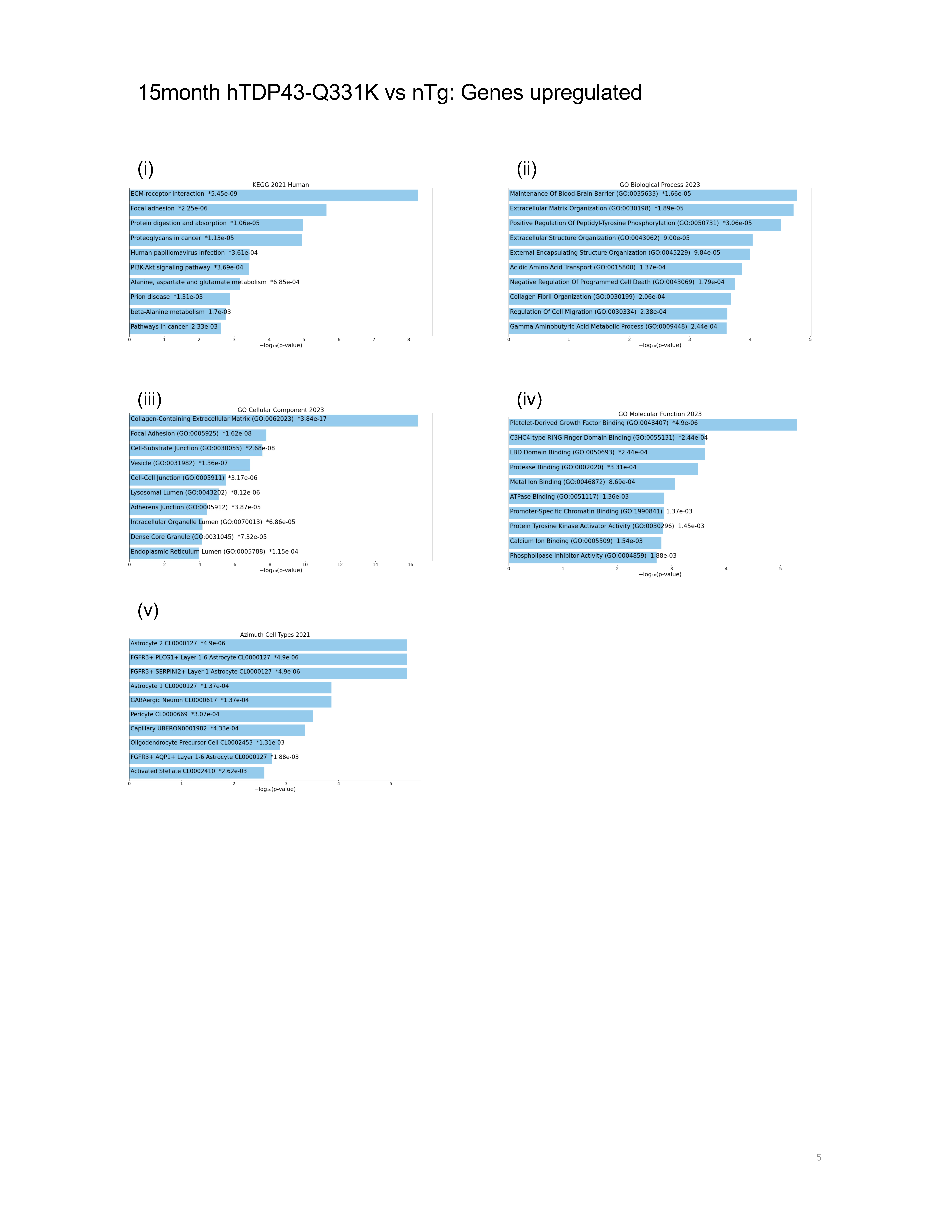

Supplement: S5 Fig — In the 15 month-old hTDP-43-Q331K MC, differentially expressed transcripts were enriched for ECM receptor interaction, focal adhesion, and PI3K-Akt signaling pathway genes. Additional upregulation was found in genes associated with ECM organization and peptidyl-tyrosine phosphorylation. There was also a significant increase in the expression of genes specific to layer 1–6 astrocytes and GABAergic neurons. X- axis represents -log10(Padj) values for each component. Figures generated on Enrichr [87–89]. (TIF) [file pgen.1012007.s005.tif]

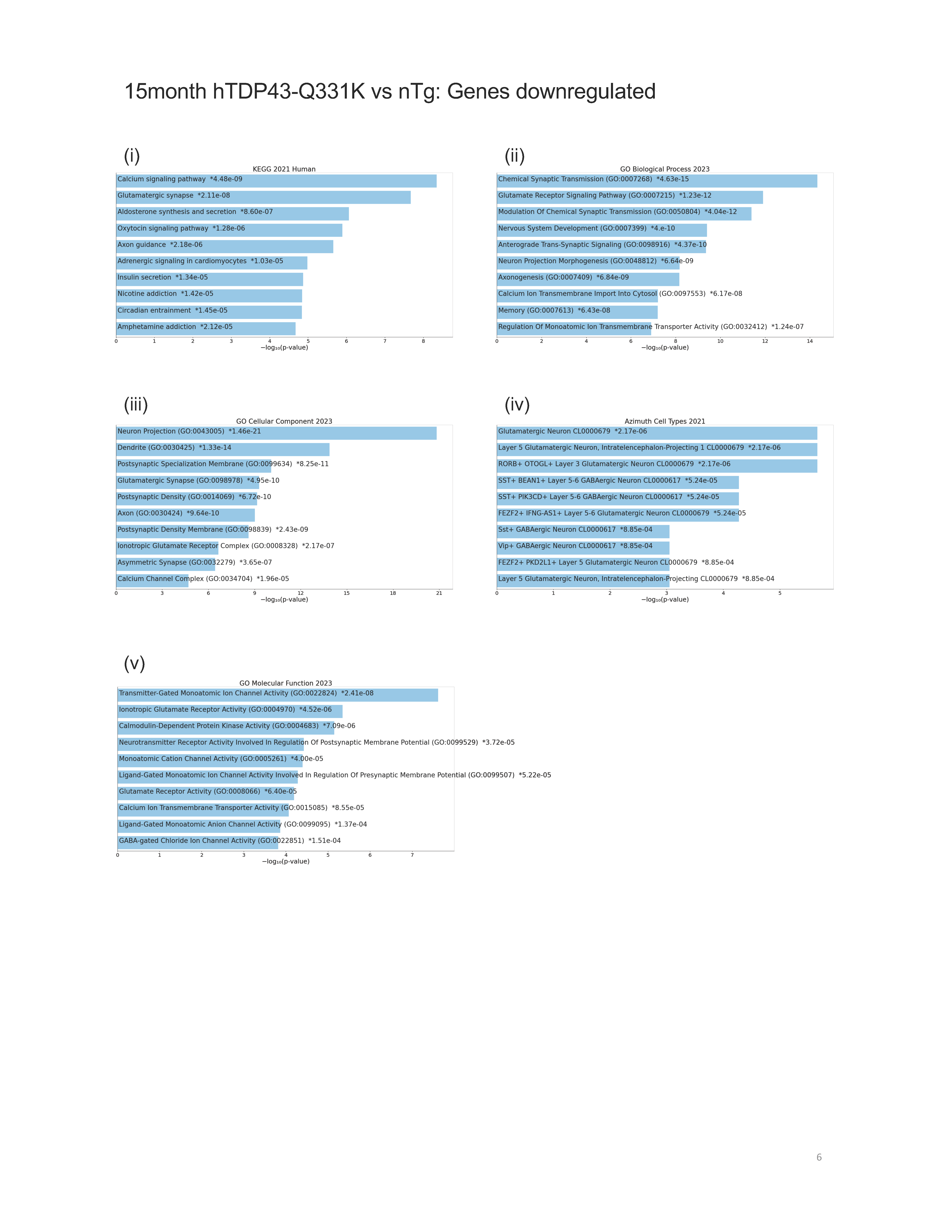

Supplement: S6 Fig — Genes involved in the calcium signaling pathway, glutamatergic synapses, glutamate receptor signaling pathway, and nervous system development were downregulated. This downregulation extended to genes expressed in both glutamatergic and GABAergic neurons. X- axis represents -log10(Padj) values for each component. Figures generated on Enrichr [87–89]. (TIF) [file pgen.1012007.s006.tif]

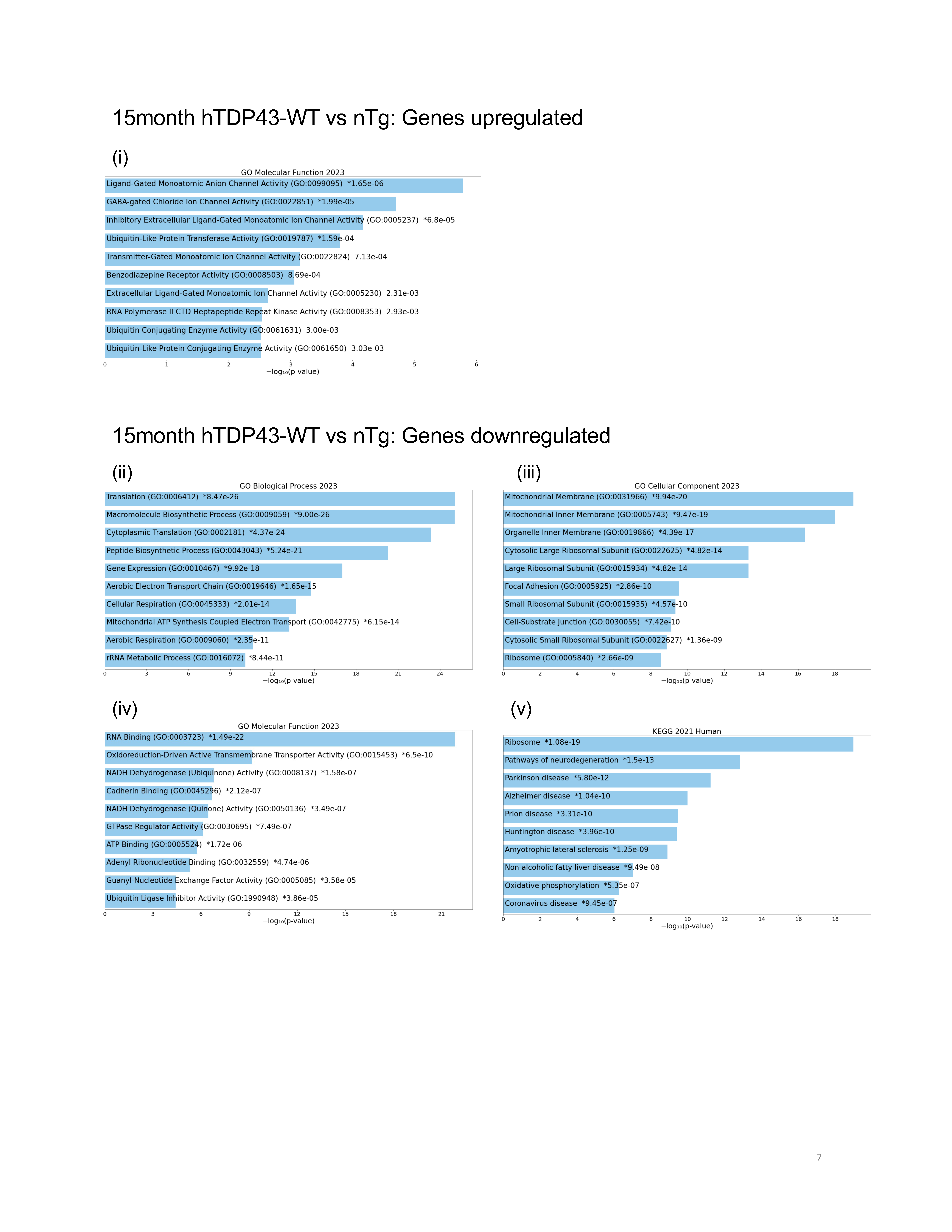

Supplement: S7 Fig — In the 15 month-old hTDP-43-WT MC, differentially expressed genes were enriched for those associated with GABA-gated chloride ion channel activity and ubiquitin-like protein transferase activity. Additionally, these genes were depleted in gene sets associated with translation, macromolecule biosynthesis, and rRNA metabolism. Genes related to RNA binding, active transmembrane transporter activity, GTPase regulator activity, and ubiquitin ligase inhibitor activity were also significantly downregulated. X- axis represents -log10(Padj) values for each component. Figures generated on Enrichr [87–89]. (TIF) [file pgen.1012007.s007.tif]

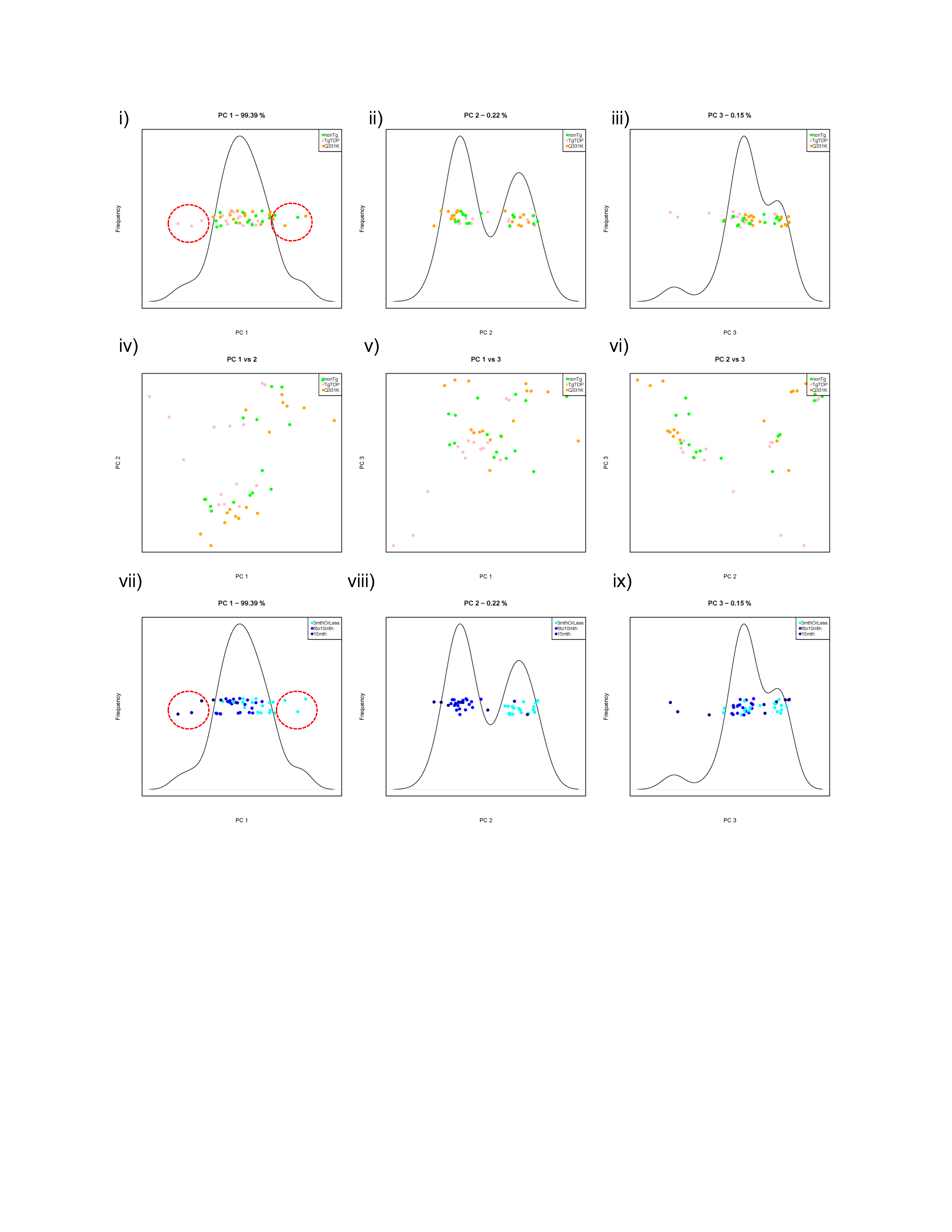

Supplement: S8 Fig — (TIF) [file pgen.1012007.s008.tif]

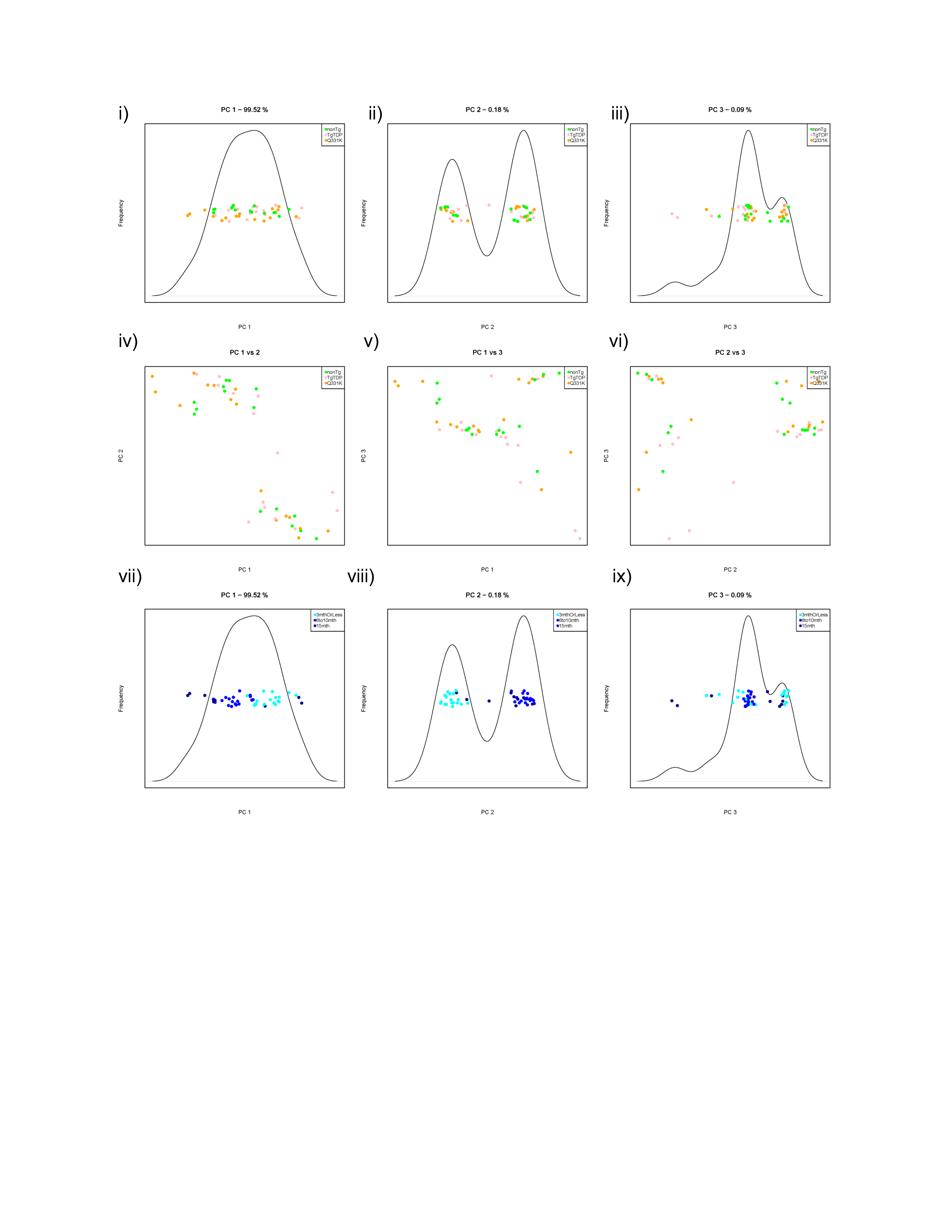

Supplement: S9 Fig — (TIF) [file pgen.1012007.s009.tif]

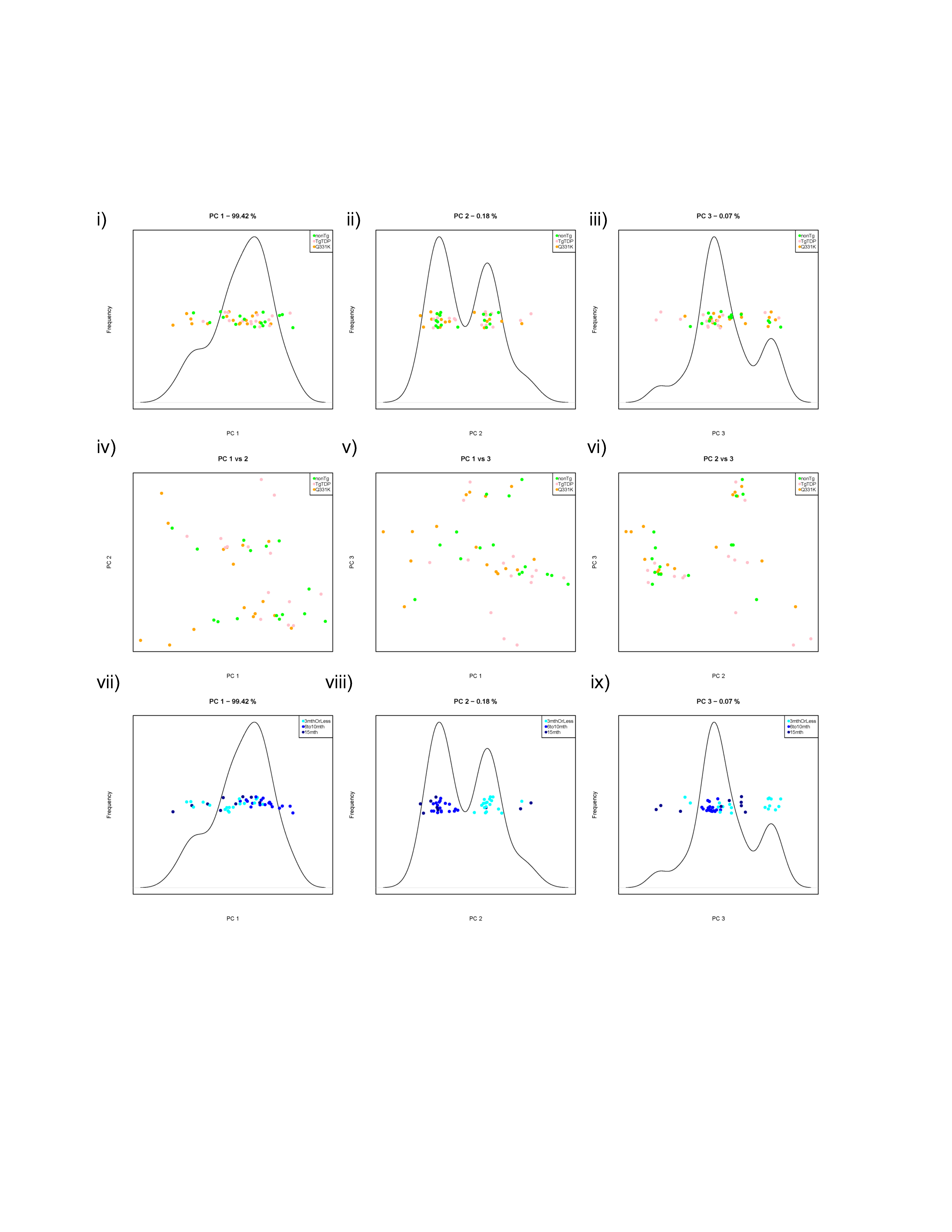

Supplement: S10 Fig — (TIF) [file pgen.1012007.s010.tif]

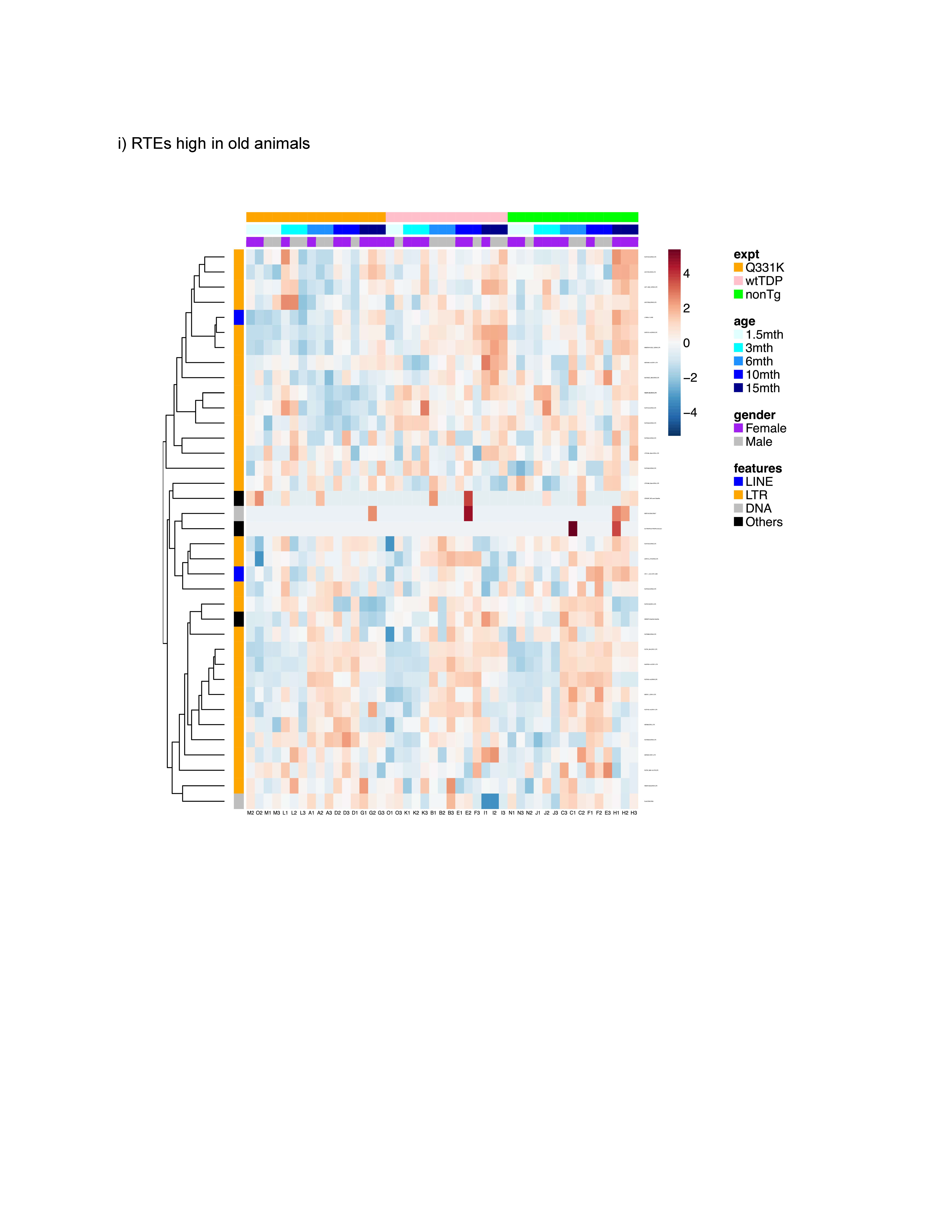

Supplement: S12 Fig — i) Heatmap showing expression of RTEs known to be highly expressed in old animals at 1.5-, 3-, 6-, 10- and 15-month time points in the MC total RNA of hTDP-43-Q331K, hTDP-43-WT and non-transgenic littermates. We note that RTEs that exhibit high expression later in life in control animals are not significantly expressed in the 15-month-old hTDP43-WT animals (padj = 7.31E-04, odds ratio = 0.249). (TIF) [file pgen.1012007.s012.tif]

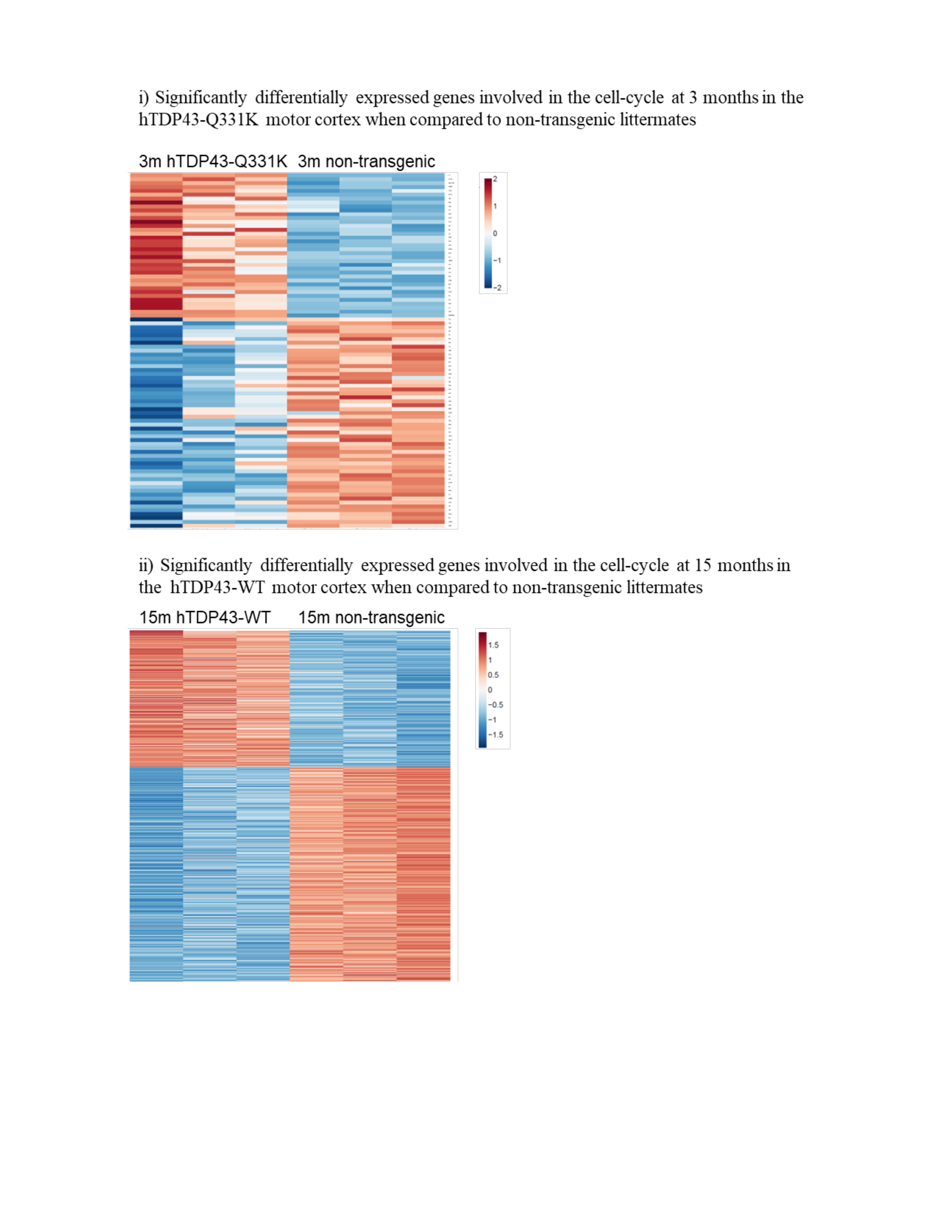

Supplement: S13 Fig — i) Heatmap showing significantly differentially expressed genes involved in the cell cycle at 3 months in the MC total RNA of hTDP-43-Q331K, and non-transgenic littermates. ii) Heatmap showing significantly differentially expressed genes involved in the cell cycle at 15 months in the MC total RNA of hTDP-43-WT and non-transgenic littermates. (TIF) [file pgen.1012007.s013.tif]

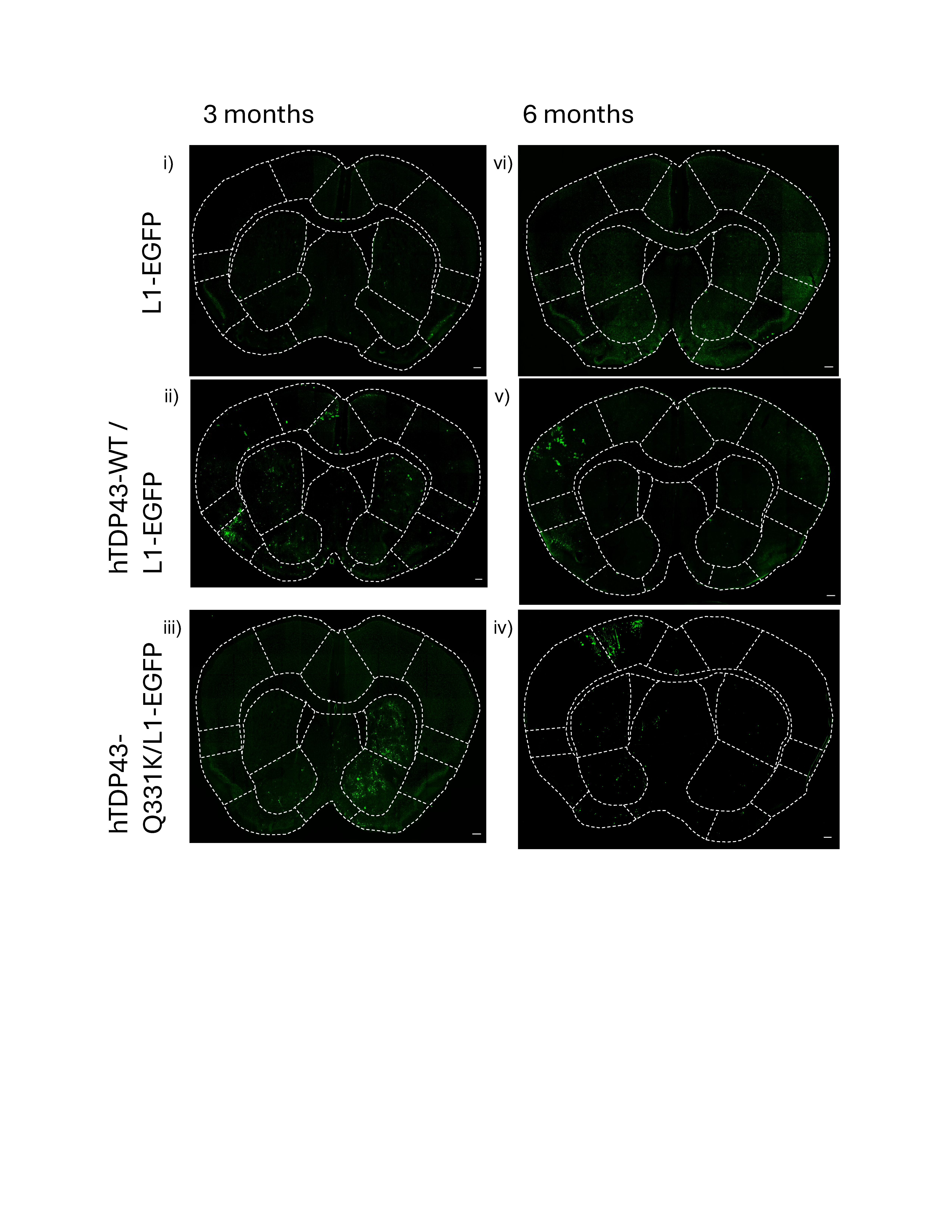

Supplement: S14 Fig — i) Representative whole brain images from 3- and 6-month-old L1-EGFP, hTDP-43-WT/L1-EGFP and hTDP-43-Q331K/L1-EGFP animals showing LINE-1 retrotransposition clusters in different regions such as the striatum, nucleus accumbens, agranular insular cortex, and piriform cortex. Scalebar = 100μm. (TIF) [file pgen.1012007.s014.tif]

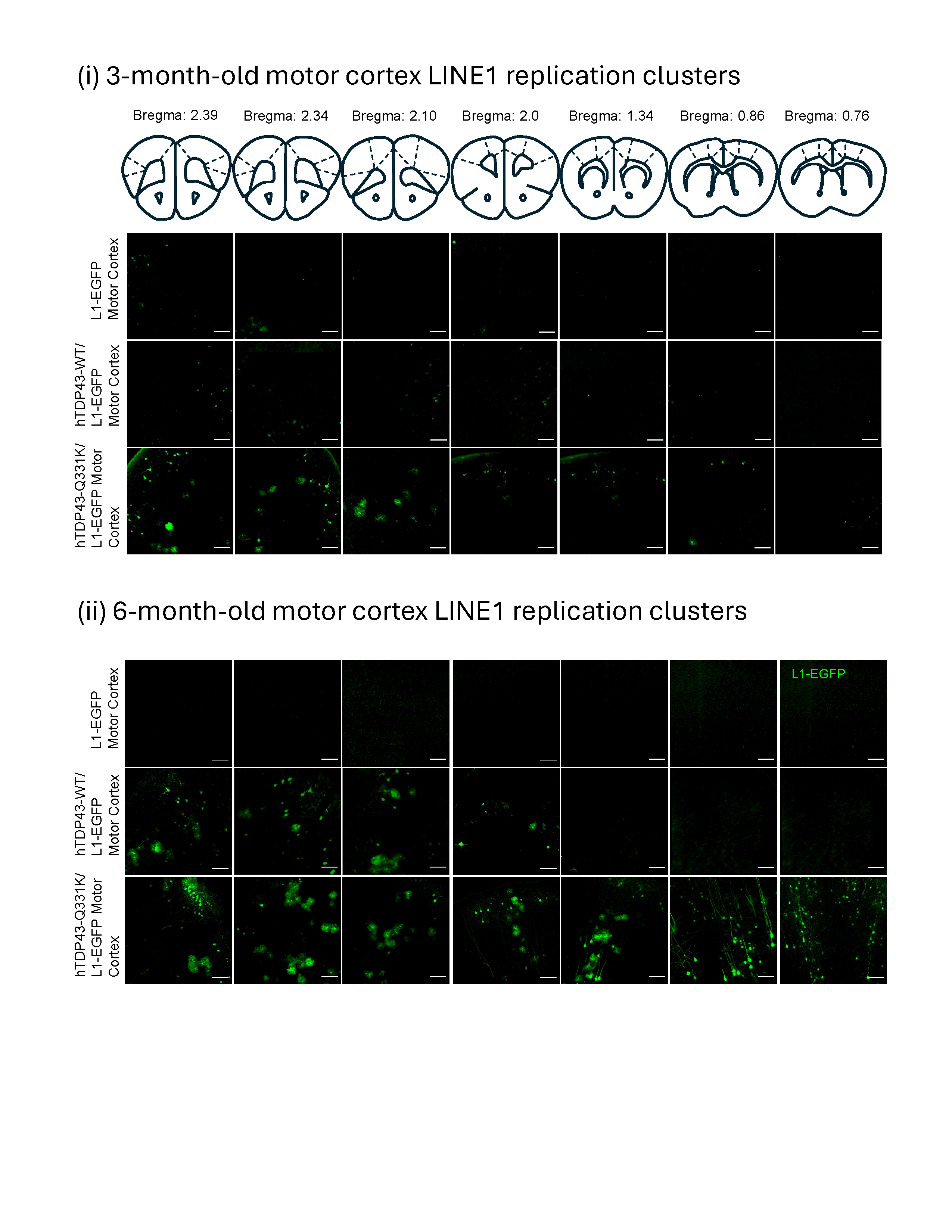

Supplement: S15 Fig — Scalebar = 50μm. Bregma images created in Powerpoint. (TIF) [file pgen.1012007.s015.tif]

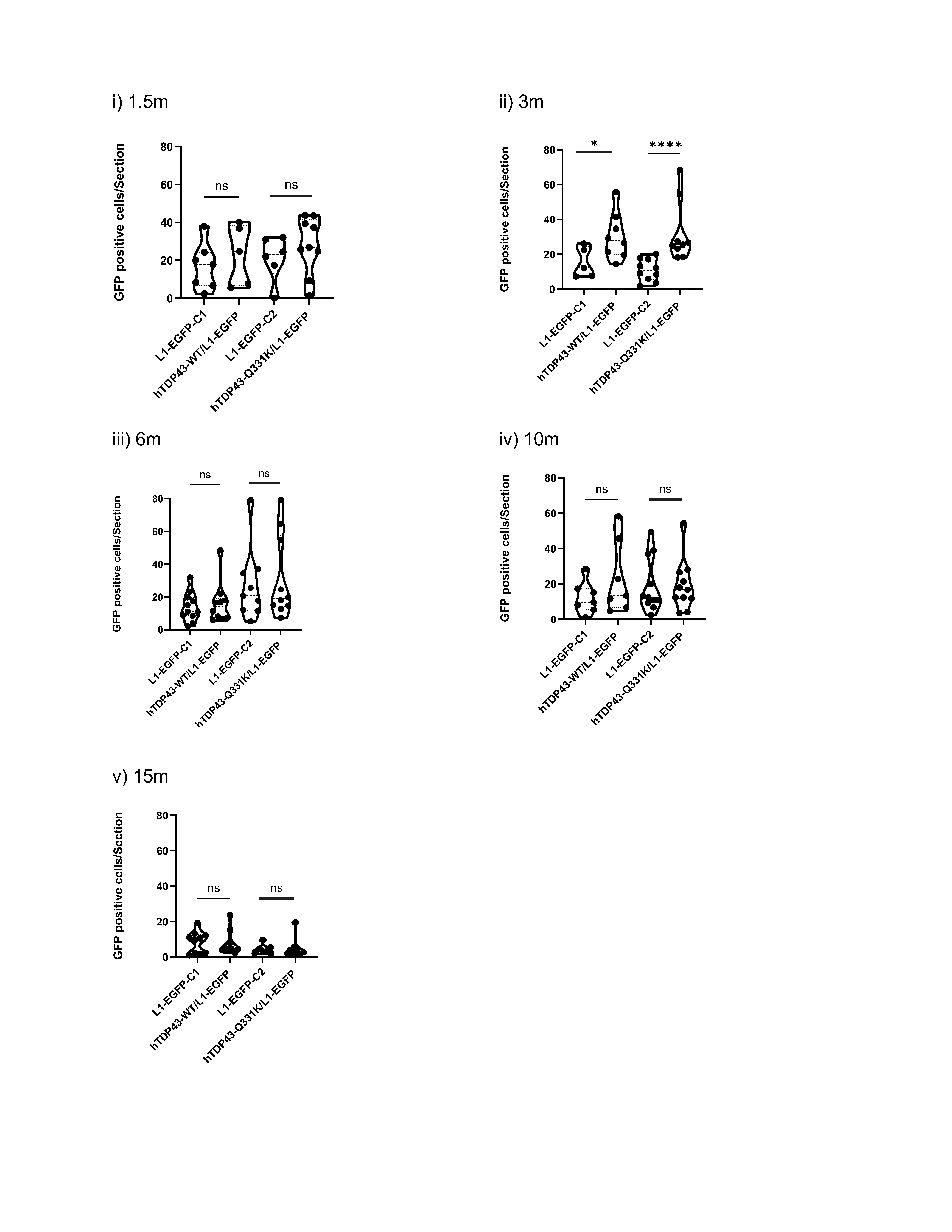

Supplement: S16 Fig — (a-e) GFP positive cells/ section in 1.5-, 3-, 6-, 10-, and 15-m, respectively. Images were processed on FIJI- ImageJ and manually counted. Mann- Whitney test was done to compute the significance with * p ≤ 0.05 and ** p < 0.01. p-value for (ii) was 0.0451 and <0.0001, respectively. Mixed cohorts of n = 5–11 animals were used for all genotypes and age groups. hTDP-43-WT/L1-EGFP mice and hTDP-43-Q331K/L1-EGFP mice were maintained as separate colonies and hence have been compared to their littermates L1-EGFP-C1 and L1-EGFP-C2, respectively. (TIF) [file pgen.1012007.s016.tif]

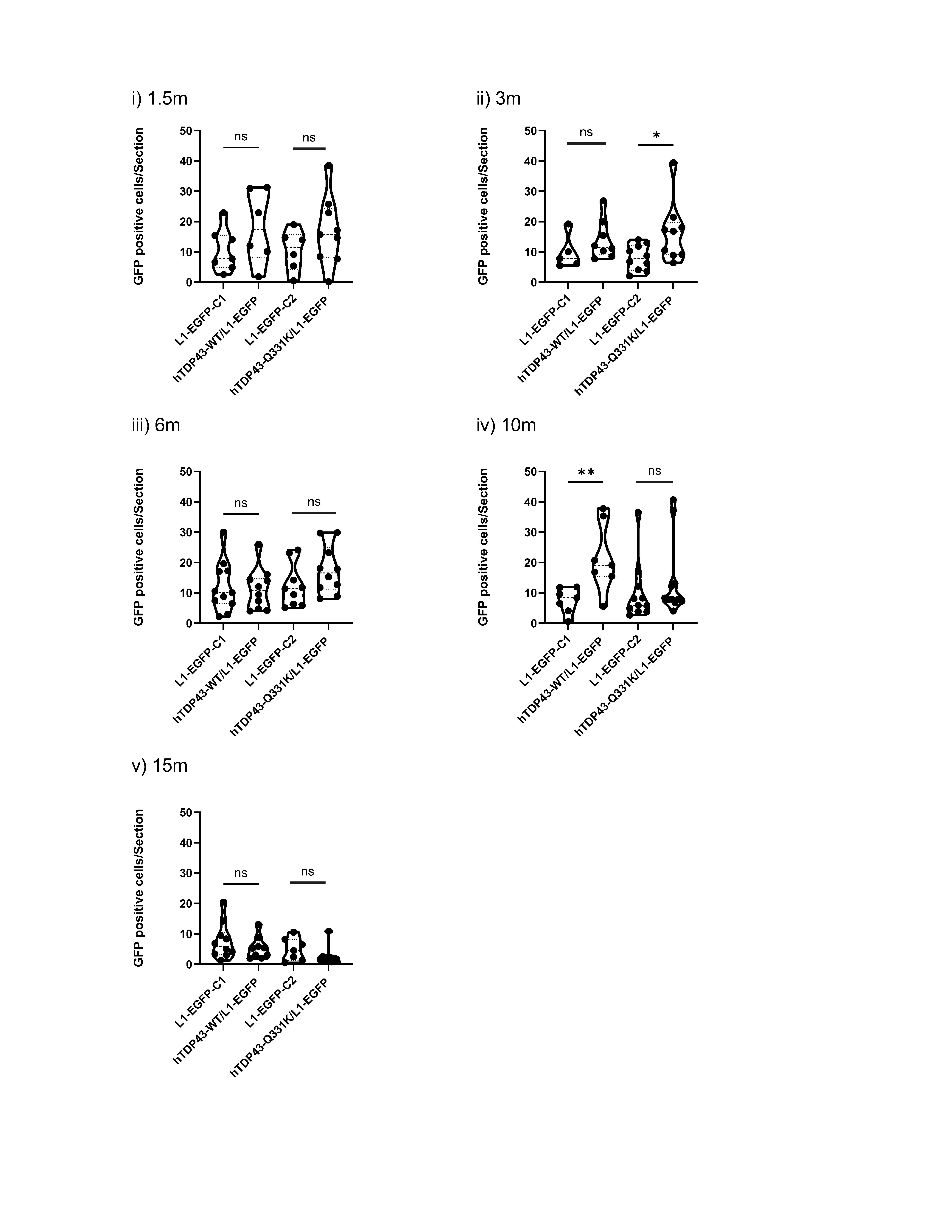

Supplement: S17 Fig — (i-v) GFP positive cells/ section in 1.5 m, 3m, 6m, 10m, and 15m, respectively. Images were processed on FIJI- ImageJ and manually counted. Mann- Whitney test unpaired t-test was done to compute the significance with * p ≤ 0.05 and ** p < 0.01. p-value for (ii) was 0.0220 and for (iv) was 0.0098respectively. Mixed cohorts of n = 5–11 animals were used for all genotypes and age groups. hTDP-43-WT/L1-EGFP mice and hTDP-43-Q331K/L1-EGFP mice were maintained as separate colonies and hence have been compared to their littermates L1-EGFP-C1 and L1-EGFP-C2, respectively. (TIF) [file pgen.1012007.s017.tif]

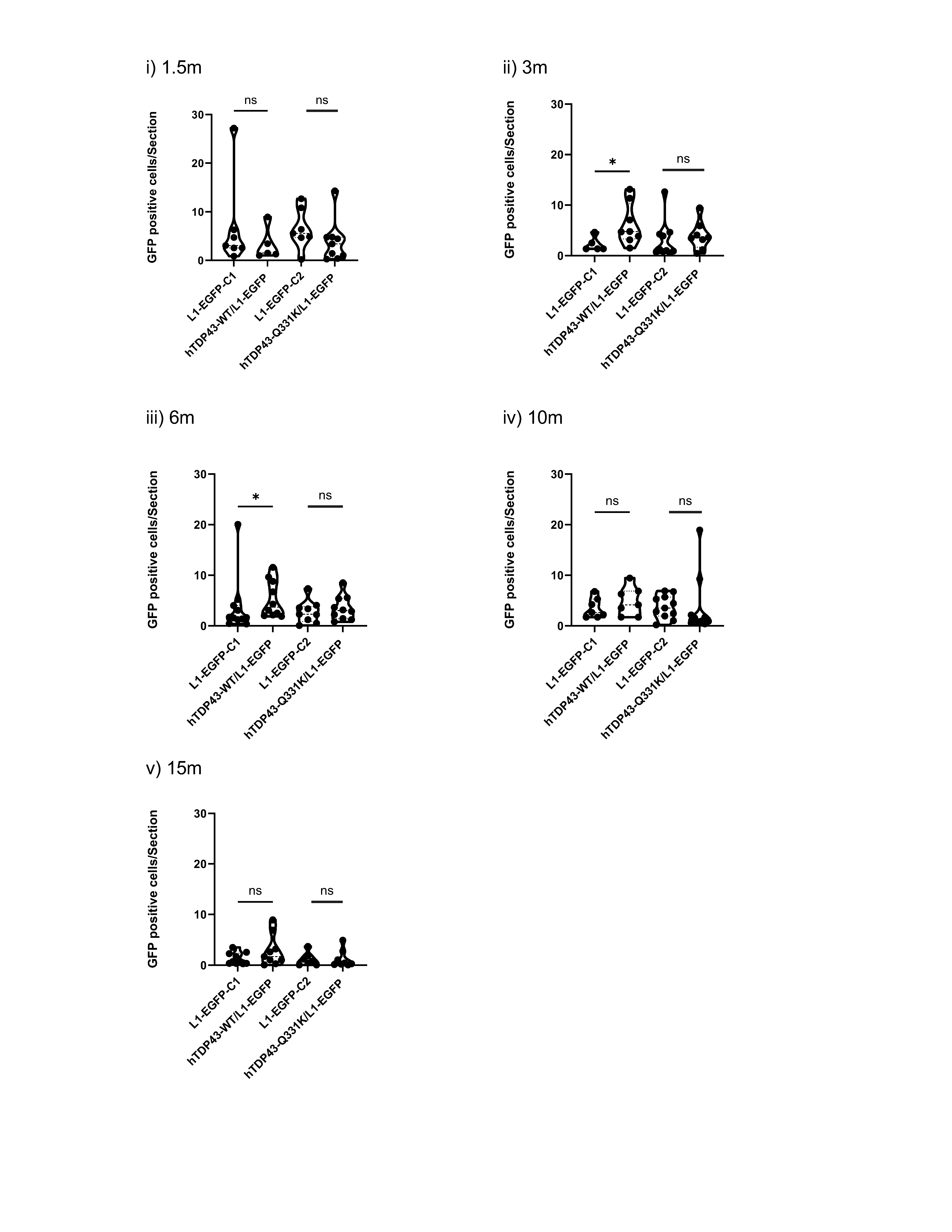

Supplement: S18 Fig — (i-v) GFP positive cells/ section in 1.5 m, 3m, 6m, 10m, and 15m, respectively. Images were processed on FIJI- ImageJ and manually counted. Mann- Whitney test unpaired t-test was done to compute the significance with * p ≤ 0.05 and ** p < 0.01. p-value for (ii) was 0.0186 and for (iii) was 0.0375. Mixed cohorts of n = 5–11 animals were used for all genotypes and age groups. hTDP-43-WT/L1-EGFP mice and hTDP-43-Q331K/L1-EGFP mice were maintained as separate colonies and hence have been compared to their littermates L1-EGFP-C1 and L1-EGFP-C2, respectively. (TIF) [file pgen.1012007.s018.tif]
