# Supplementary material for: LINE-1 retrotransposition in a mouse TDP-43 model of neurodegeneration marks motor cortex neurons for cell-intrinsic and cell non-autonomous programmed cell death
Source: PLoS Genet. 2025 Dec 29;21(12):e1012007. doi: 10.1371/journal.pgen.1012007 (PMC12758826; doi:10.1371/journal.pgen.1012007)
Supplement: Attachment — Submitted filename: Comments_July 2025.pdf [file pgen.1012007.s034.pdf]

The authors investigated the activation of RTEs and ERVs in wild-type mice and mice expressing wild-type or Q331K mutant hTDP-43 across different ages. The authors further crossed these lines with a human LINE-1 EGFP reporter strain to assess LINE-1 retrotransposition events across age. The authors report transient changes in RTE and ERV transcript levels in hTDP-43-WT mice at 15mo and in hTDP-43-Q331K mice at 3mo of age. These time points correspond to the onset or pre-onset of behavioral phenotypes. These findings suggest that RTE/ERV activation is temporally restricted and not persist with disease progression in hTDP-43 mouse model, providing insight into when targeting these elements might be therapeutically relevant. This is an interesting and timely study. However, several concerns should be addressed or clarified.

Major Concerns:

Please clarify the inclusion and exclusion criteria for the motor behavioral tests. Were animals excluded based on performance, general health, or genotype-related severity? The number of mice used for hindlimb clasping test in the hTDP-43-WT group differs between time points (n=6 at 10mo vs. n=10 at 15mo). Please explain the rationale for this discrepancy.

In Fig. 1g and 1m, there is no hTDP-43-Q331K group shown at 17mo. Was this due to reduced survival or other experimental limitations?

Mouse sex information for behavioral testing. Were males, females, or a combination used? Were any sex-specific effects observed?

From Fig. 2c, it appears that hTDP-43-WT mice at 15mo exhibit both higher expression levels and a greater number of significantly differentially expressed RTEs compared to hTDP-43-Q331K mice at 3mo. Have the authors directly compared these two groups? Given that both represent early onset or initial stages of motor deficits, such a comparison could provide valuable insight into whether WT or mutant hTDP-43 has a stronger impact on RTE expression.

Page 10, lines 227–239: It would strengthen the manuscript to clarify which specific RTE/ERV families show age-dependent expression changes, and which are elevated in the hTDP-43 mouse models. Are these two sets overlapping or distinct?

Although hTDP-43-Q331K mice do not show significantly elevated LINE-1 transcript levels at 3mo and show reduced levels at 6mo (Fig 2c), an increase in LINE-1 EGFP reporter-based retrotransposition events is observed (Figs 3-4). An increased retrotransposition typically is correlated with increased LINE-1 transcript levels. To reconcile this discrepancy, the authors should provide a mechanistic explanation or quantify LINE-1 DNA copy number in the same samples to validate that actual retrotransposition occurred, not just reporter expression.

Page 15, lines 469–494: The authors report that the number of TUNEL-positive neurons with LINE-1 reporter activity are not significantly different across three genotypes, suggesting that neuron cell death is independent of hTDP-43 genotype. However, in lines 469–470, they also state that hTDP-43-driven LINE-1 retrotransposition in neurons predicts cell-intrinsic death. Please clarify whether LINE-1 activation in neurons is predictive of cell death in this model or not.

Minor Concerns:

Fig. 1g and 1m: The figure panels are not aligned in size with the rest of Fig. 1. Please adjust for consistency.

Fig. 2a: The hTDP-43-WT group at 1.5mo appears to show only two columns in the heatmap (n=2). It appears across all heatmaps. Is this correct? The methods indicate n=3 for each group.

The relationship between Fig. 2a and Supplementary Fig. 2ai is unclear. Both appear to show differential RTE expression across ages in three mouse models. Please clarify if there is any difference in dataset or analysis. Similar ambiguity also applies to several other supplementary figures.

Several paragraphs are somewhat repetitive and would improve clarity and conciseness.

Page 19, line 639: Please correct “IUCAC” to “IACUC.”

Page 22, lines 697–715: Please indicate which figure this paragraph corresponds to.
