# Supplementary material for: LINE-1 retrotransposition in a mouse TDP-43 model of neurodegeneration marks motor cortex neurons for cell-intrinsic and cell non-autonomous programmed cell death
Source: PLoS Genet. 2025 Dec 29;21(12):e1012007. doi: 10.1371/journal.pgen.1012007 (PMC12758826; doi:10.1371/journal.pgen.1012007)
Supplement: Attachment — Submitted filename: Response to Reviewers.pdf [file pgen.1012007.s036.pdf]

We wish to thank the reviewers for their insightful and helpful comments. In our revised manuscript, we have endeavored to fully address all reviewer concerns.

Journal Requirements:
1) We ask that a manuscript source file is provided at Revision. Please upload your manuscript file as a .doc, .docx, .rtf or .tex. If you are providing a .tex file, please upload it under the item type u2018LaTeX Source Fileu2019 and leave your .pdf version as the item type u2018Manuscriptu2019.

- **We have provided the requested files.**

2) Please upload all main figures as separate Figure files in .tif or .eps format. For more information about how to convert and format your figure files please see our guidelines: https://protect.checkpoint.com/v2/r01/___https://journals.plos.org/plosgenetics/s/figures___.YzJ1OnN0b255YnJvb2s6YzpnOjA5NGRkMzkyNjdlYzAyYWJmZTYwYmM5NDVjZjRmMTFmOjc6ZjRhZDphZTI2ZDQ5M2JjZDFkNzE2MDQzYTYzZjIxOTE5NDFhNTllNDJhZDZmMDI3MGNlNjczMmZjYjgzYTZhNTFlNTRmOnQ6VDpG

- **We have provided the required figure files in .tif formats.**

3) Some material included in your submission may be copyrighted. According to PLOSu2019s copyright policy, authors who use figures or other material (e.g., graphics, clipart, maps) from another author or copyright holder must demonstrate or obtain permission to publish this material under the Creative Commons Attribution 4.0 International (CC BY 4.0) License used by PLOS journals. Please closely review the details of PLOSu2019s copyright requirements here: PLOS Licenses and Copyright. If you need to request permissions from a copyright holder, you may use PLOS's Copyright Content Permission form. Please respond directly to this email and provide any known details concerning your material's license terms and permissions required for reuse, even if you have not yet obtained copyright permissions or are unsure of your material's copyright compatibility. Once you have responded and addressed all other outstanding technical requirements, you may resubmit your manuscript within Editorial Manager.
Potential Copyright Issues:

i) Please confirm (a) that you are the photographer of 1A, or (b) provide written permission from the photographer to publish the photo(s) under our CC BY 4.0 license.

RESPONSE: Korada is the photographer for 1A. It is a frame from one of the hindlimb clasping videos. The rotarod is a biorender premium account image. We have sent the license pdf previously and have uploaded it as an "other" file in the resubmission.

ii) Figures 1, 4a, 6a, and S3b. Please confirm whether you drew the images / clip-art within the figure panels by hand. If you did not draw the images, please provide (a) a link to the source of the images or icons and their license / terms of use; or (b) written permission from the copyright holder to publish the images or icons under our CC BY 4.0 license. Alternatively, you may replace the images with open source alternatives. See these open source resources you may use to replace images / clip-art:
-
https://protect.checkpoint.com/v2/r01/___https://commons.wikimedia.org___.YzJ1OnN0b255YnJvb2s6YzpnOjA5NGRkMzkyNjdlYzAyYWJmZTYwYmM5NDVjZjRmMTFmOjc6NzE4OTowNjA3YWIyZDBkODI4NTUzNzdmNzdiYTJhMDg1NThiODFkN2E0NjcxMGU3MDY5OTQ3MzBiNDBiMWViMGY5N2VjOnQ6VDpG
-
https://protect.checkpoint.com/v2/r01/___https://openclipart.org/___.YzJ1OnN0b255YnJvb2s6YzpnOjA5NGRkMzkyNjdlYzAyYWJmZTYwYmM5NDVjZjRmMTFmOjc6ODk4MDphY2JjNzZiOWRmYzBmZTdmMTE1OWVkYmU0ZTVkZjdiMDY3ZWQ3ZDBmYmE3NGJkYjE5M2U2NmI4MGFlYzBiNWRiOnQ6VDpG.

RESPONSE:

Fig 1: 1a- the mice were photographed by Korada. The rotarod is a bioRender image.

Fig 4a: The original brain maps have been replaced with new brain maps created by us in powerpoint. The IHC images were photographed by Korada.

Fig 6a: The original brain maps have been replaced with new brain maps created by us in powerpoint. The lateralization map was built by Korada.

Fig S3b: The original brain maps have been replaced with new brain maps created by us in powerpoint. The IHC images were photographed by Korada.

4) Please amend your detailed Financial Disclosure statement. This is published with the article. It must therefore be completed in full sentences and contain the exact wording you wish to be published.
1) State what role the funders took in the study. If the funders had no role in your study, please state: "The funders had no role in study design, data collection and analysis, decision to publish, or preparation of the manuscript."
2) If any authors received a salary from any of your funders, please state which authors and which funders.

- **We have amended the financial disclosure statement as requested.**

5) Please ensure that the funders and grant numbers match between the Financial Disclosure field and the Funding Information tab in your submission form. Note that the funders must be provided in the same order in both places as well. Currently, the order of the funders are not the same in both places.

- **We have ensured that all funders and grant numbers match.**

Specific Reviewer Critiques:

Reviewer #1: The authors investigated the activation of RTEs and ERVs in wild-type mice and mice expressing wild-type or Q331K mutant hTDP-43 across different ages. The authors further crossed these lines with a human LINE-1 EGFP reporter strain to assess LINE-1 retrotransposition events across age. The authors report transient changes in RTE and ERV transcript levels in hTDP-43-WT mice at 15mo and in hTDP-43-Q331K mice at 3mo of age. These time points correspond to the onset or pre-onset of behavioral phenotypes. These findings suggest that RTE/ERV activation is temporally restricted and not persist with disease progression in hTDP-43 mouse model, providing insight into when targeting these elements might be therapeutically relevant. This is an interesting and timely study. However, several concerns should be addressed or clarified.

Major Concerns:

- Please clarify the inclusion and exclusion criteria for the motor behavioral tests. Were animals excluded based on performance, general health, or genotype-related severity? The number of mice used for hindlimb clasping test in the hTDP-43-WT group differs between time points (n=6 at 10mo vs. n=10 at 15mo). Please explain the rationale for this discrepancy.

  RESPONSE: Animals were not excluded based on any criteria. n=6 to 12 was used for all groups depending on availability of animals. The groups with lower n were due to breeding efficiency and colony maintenance issues. Since the time of submission, were able to increase the n to 7 for the hTDP-43-WT group at 10 months and have modified the figure/ statistics to reflect that.

- In Fig. 1g and 1m, there is no hTDP-43-Q331K group shown at 17mo. Was this due to reduced survival or other experimental limitations?

  RESPONSE: We thank the reviewer for mentioning this. This was in fact due to reduced survival of the Q331K line and an inability to obtain enough mice at that age.

- Mouse sex information for behavioral testing. Were males, females, or a combination used? Were any sex-specific effects observed?

  RESPONSE: Mixed-sex cohorts were used for all experiments including behavior. We could not detect any sex-specific effects in the hTDP-43-Q331K or the hTDP-43-WT mice.

- From Fig. 2c, it appears that hTDP-43-WT mice at 15mo exhibit both higher expression levels and a greater number of significantly differentially expressed RTEs compared to hTDP-43-Q331K mice at 3mo. Have the authors directly compared these two groups? Given that both represent early onset or initial stages of motor deficits, such a comparison could provide valuable insight into whether WT or mutant hTDP-43 has a stronger impact on RTE expression.

  RESPONSE: The reviewer is correct on both counts. We have directly compared the differential expression of TEs in the 3mo hTDP-43-Q331K and the 15mo hTDP-43-WT and we found 229 TEs that were significantly differentially expressed. These TEs include LINEs, LTRs, SINEs, and DNA transposons. While 104 of these TEs were significantly upregulated in the 3mo hTDP-43-Q331K in comparison to the 15mo hTDP-43-WT animals, 125 were downregulated. Thus, a direct comparison doesn't tell us which transgene has a stronger impact on RTE expression. On the other hand, it is true that there are more differentially expressed RTEs in the TDP-43-wt vs control littermate at 15 months than in the TDP-43-Q331K vs control littermates at 3 months. We think that a likely explanation for this is that there may be an increased rate of cell death in

response to RTE expression in the TDP-43-Q331K transgenic animals. But we have no direct evidence to address this.

- Page 10, lines 227–239: It would strengthen the manuscript to clarify which specific RTE/ERV families show age- dependent expression changes, and which are elevated in the hTDP-43 mouse models. Are these two sets overlapping or distinct?

RESPONSE: The RTEs that are upregulated with age do not overlap significantly with the RTEs upregulated in the hTDP43 mouse models. The RTEs that are upregulated with age are made up entirely of LTR, SINE, and DNA transposons. They do not show significant upregulation of any LINE family elements. However, the retrotransposon families upregulated in the hTDP-43 mouse models significantly overlap with those that are normally expressed in MC of young animals, and these include LTRs, DNA transposons, SINEs and LINEs. The LINE family elements are significantly upregulated are exclusively at the earlier time points, or upon expression of hTDP-43 transgenes, but not with aging.

- Although hTDP-43-Q331K mice do not show significantly elevated LINE-1 transcript levels at 3mo and show reduced levels at 6mo (Fig 2c), an increase in LINE-1 EGFP reporter-based retrotransposition events is observed (Figs 3-4). An increased retrotransposition typically is correlated with increased LINE-1 transcript levels. To reconcile this discrepancy, the authors should provide a mechanistic explanation or quantify LINE-1 DNA copy number in the same samples to validate that actual retrotransposition occurred, not just reporter expression.

  RESPONSE: The LINE-1 retrotransposition events are rare in the healthy controls but show up as clusters of cells in the motor cortex in the hTDP-43-Q331K and hTDP-43-WT transgenic animals. We also observe that the cells that exhibit LINE-1 replication are co-labelled with TUNEL, indicating that they are undergoing cell death. For these reasons, the fraction of cells that would be predicted to exhibit LINE-1 expression is likely quite low at any given moment. Despite this, we are able to detect increased expression of some LINE-1 elements in the bulk seq at some timepoints. But the reviewer's point is a good one, the lack of detection of a given RTE as being differentially expressed in any given timepoint cannot be taken as evidence that it is not differentially expressed in some cells. In our view, this sparse expression is the most likely explanation for why we do not detect LINE-1 expression in the 3-month bulk RNA-Seq experiments. We also appreciate the reviewer's suggestion to measure LINE-1 DNA copy number. We also have thought about this, but we do not think this is likely to work for several reasons. First, the total copy number of LINE-1 elements in the genome is very high-many thousands of elements per diploid genome. Thus, even within the individual cells where LINE-1 replication events take place, the expectation is that the total number of elements would only change by a tiny fraction. This issue is compounded by the fact that only a small fraction of cells exhibits LINE-1 replication events.

- Page 15, lines 469–494: The authors report that the number of TUNEL-positive neurons with LINE-1 reporter activity are not significantly different across three genotypes, suggesting that neuron cell death is independent of hTDP-43 genotype. However, in lines 469–470, they also state that hTDP-43-driven LINE-1 retrotransposition in neurons predicts cell-intrinsic death. Please clarify whether LINE-1 activation in neurons is predictive of

cell death in this model or not.

RESPONSE: We thank the reviewer for this comment, which makes us realize that we were unclear in how we described the findings. The finding is that when a neuron is LINE-l-eGFP positive, it is also highly likely to be TUNEL positive irrespective of the genotype of the animals. But as shown in Fig 3 and 4, such LINE-1 replication events rarely occur in the motor cortex of the healthy controls, but occur far more frequently in the presence of the hTDP-43-WT or hTDP-43-Q331K transgene. This leads to a higher number of TUNEL labeled neurons in the presence of the TDP-43 transgenes. The other finding that we report is that the increased rate of TUNEL label in cells that are nearby to LINE-1-eGFP positive neurons only occurs in the context of TDP-43 expression.

Minor Concerns:

- Fig. 1g and 1m: The figure panels are not aligned in size with the rest of Fig. 1. Please adjust for consistency.

  RESPONSE: We have adjusted those figures as requested.

- Fig. 2a: The hTDP-43-WT group at 1.5mo appears to show only two columns in the heatmap (n=2). It appears across all heatmaps. Is this correct? The methods indicate n=3 for each group.

  RESPONSE: The figure legend of Fig 2 mentions that "n=3 mixed-sex, age-matched cohorts were used for all genotypes and age groups except 1.5 month, where n=2 for nTg and n=4 for hTDP-43-WT Tg were used. See Methods for details regarding the analysis pipeline, including statistical analyses."

- The relationship between Fig. 2a and Supplementary Fig. 2ai is unclear. Both appear to show differential RTE expression across ages in three mouse models. Please clarify if there is any difference in dataset or analysis. Similar ambiguity also applies to several other supplementary figures.

  RESPONSE: Fig 2a shows only significantly differentially expressed RTE while Supplementary Fig 2ai shows expression levels of all TEs. These corrections are now included in the figure legends and titles.

- Several paragraphs are somewhat repetitive and would improve clarity and conciseness.

RESPONSE: We thank the reviewers for noticing this and we have worked to create a more concise manuscript.

- Page 19, line 639: Please correct "IUCAC" to "IACUC."

RESPONSE: This has been corrected.

- Page 22, lines 697–715: Please indicate which figure this paragraph corresponds to.

RESPONSE: This has been corrected. It was Supplemental Table 2d.

Reviewer #2: Korada and colleagues investigate the role of TDP-43 in repressing retrotranspon elements (RTEs) using an established two mouse models of TDP-43 pathology, the mild overexpression of wildtype or Q331K human TDP-43. They show that the Q331K mouse has early motor deficits compared to a later deficit in the WT. They cross the mice to a LINE1 GFP reporter line to observe LINE1 re-integration in the motor cortex. They show that this LINE1 integration is enhanced at particular time points in the TDP-43 mutant lines, and that this is accompanied by markers of cell death in LINE1-positive neurons, along with nearby cells.

This is a very interesting paper for the field, showing that TDP-43 is associated with LINE de-repression and suggesting that this de-repression is killing both neurons and neighboring cells. Overall, the paper is well written and the methods are clearly described. I have few major comments, but would suggest that the figures are improved to be more legible and consistent across panels.

Major comment: are the authors planning to look at TDP-43 dependent splicing changes in their RNA-seq data? It would be useful to show the accumulation of both loss-of-function (cryptic exons) and gain of function (skiptic exons) at different time points to see whether these splicing changes co-occur with the RTE changes observed. There is a database of mouse TDP-43 splicing events in Fratta et al, 2018 (PMID: 29764981) that could be queried.

RESPONSE: We thank the reviewer for making this comment and have mentioned the original splicing defect results in the last paragraph of the introduction. The original manuscript describing the development of these mouse models (Arnold 2013 PNAS) did in fact describe extensive TDP-43 relevant splicing defects in both lines of these animals, despite a lack of nuclear loss or cytoplasmic accumulation of TDP43. This finding from the 2013 study establishes that these models do reflect loss of nuclear TDP43 function, leading to relevant cryptic splicing inclusion and exclusion events. Some of the examined TDP43 targets from the Arnold paper showing TDP43-relevant exon inclusion and exclusion are Eif4a, Kcnlp2, Taf1b, Abhd14a, Sort1, Kcnd3, Atxn2, and Ahl1. Such effects on splicing of TDP-43 targets were already manifested in 2 month old Q331K and w.t. transgenic animals.

Reviewer #3: This is a very nice, compact paper which makes some important discoveries that advance the field as a whole. The transient upregulation of RTEs is a significant finding. The correlation of the upregulation with onset of neurological phenotypes in the two models (WT and mutant TDP) is a significant finding. The death of neurons by apoptosis, and the “action at a distance” (non-cell autonomous death of neighboring cells) are also significant findings, and help to explain previous findings on RTE expression in human post-mortem samples. As a whole, the data are of sufficient quality to support these conclusions, and this warrants the publication of this study.

<u>Major issues</u>

While the apparent increase of L1 retrotransposition, as evidenced by the L1-EGFP reporter, is a very nice touch, given the results obtained, it also injects a certain degree of uncertainty on what’s actually going on.

1) GFP-positive “cell clusters” are seen at 3 and 6 months but not later. This correlates well with the wave of endogenous RTE expression seen in the mutant-TDP mice but not at all in the WT-TDP mice. I’m struggling to find an explanation for this. The authors should provide some speculations on this.

RESPONSE: This is a good question. While we do not have experimental evidence that clearly answers this question, we believe that differential cellular toxicity is the most likely explanation for the discrepancy in the timing of effects on RTEs in the RNA-Seq results vs the LINE-1 reporter

results for the w.t.-TDP43 mice. Our imaging experiments demonstrate TUNEL label in most of the cells that exhibit LINE-1 GFP replication, with non-cell autonomous effects on cell death also manifested. Because of the more aggressive toxicity of the Q331K transgenic animal, and the faster kinetics of LINE-1 expression and replication, there also may be differences between these genotypes in how long a cell survives once LINE-1 expression begins. We would be happy to include this speculative explanation the discussion section if the reviewer wishes. We did not include them originally precisely because they are largely speculative.

2) Relevant to the above, the L1-EGFP line is poorly defined (I looked up the Gage paper). It was made by pronuclear injection, so likely contains many copies of the reporter (at one or several genomic loci). Is anything known about this? These factors probably affect its expression and regulation. Also, how many times has the line been backcrossed to C57? The TDP models from JAX are on a C57BN background. Are the experiments in this paper done on a mixed background?

RESPONSE: We thank the reviewer for finding this lack of detail in our description of the L1-EGFP reporter line. The reviewer is correct that the L1-EGFP line was produced by pronuclear injection, which can result in many copies of the reporter, however this is in fact the SN1 line (Newkirk 2017 PNAS), which is an L1 transgene under an endogenous mouse long-interspersed nuclear element (L1) promoter, with codon-optimized mouse L1 ORF1 and ORF2 sequences, and which has been shown to have only one L1 transgene copy inserted into the first intron of the mouse tenascin R locus (Tnr) on Chromosome 1. We have added that information and citation in the mouse husbandry section of the Materials and Methods.

It is true that the TDP43 models from JAX are on a C57B6N background, and we backcrossed all three lines, including the L1-EGFP line, onto the ENVIGO C57B6N background for more than 5 generations before any intercrossing began, and we are now well more than 10 generations of backcrossing on the 6N line as we have additionally backcrossed ENVIGO 6N animals into our 3 single Tg colonies for genetic variation, with well more than 10 generations of backcrossing to 6N for all 3 lines.

3) The GFP signal tracks successful retrotransposition but not expression of the reporter itself. The L1-EGFP is human, and thus would be expressed either from its own (quite weak) promoter (inside the 5' UTR) or from a cellular promoter at the site of its insertion. I see no reason why the human L1 5'UTR would be regulated in concert with endogenous mouse L1s. Given that the authors note that the upregulation of endogenous RTEs was "similar across both evolutionarily recent and more ancient transposable elements", one possibility is that the upregulation is rather nonspecific and reflects a large-scale chromatin relaxation. It is possible that the transgene integrated in a heterochromatic region and thus could also ride this wave. In any case, it's easy to monitor the expression of the L1-EGFP reporter (it's human) by qRT-PCR, and this should be done. An alternative explanation could be that the reporter is on all the time, but that the cellular environment becomes supportive of productive retrotransposition at the observed times. It is important to address these issues because of the discrepancy noted in 1) above.

RESPONSE: Please see our previous response about this being a mouse endogenous L1 promoter model and not a human L1 promoter. We cannot distinguish whether the activation of this element in our transgenes reflects a specific activation of the promoter, or if it reflects a loss of heterochromatin. We favor the latter view for two reasons. First, we observe many different RTEs being expressed, not just LINE1. Second, there is a broader literature that includes work in human cells, human tissue and Drosophila that indicate a broad up-regulation of multiple families of RTEs/ERVS. And finally, the findings in the literature indicate that heterochromatin is in fact

changed by TDP-43 pathology. In fact, the Eddie Li lab used ATAC seq to demonstrate that LINE element chromatin is open in FTD patient neurons that lose nuclear TDP-43

4) Currently, endogenous RTE expression is monitored by RNA-seq. This is limiting for two reasons: 1) well known issues of deconvoluting expression of repetitive sequences (and assigning functional vs. non-functional L1s), 2) does not provide single-cell resolution, while much of the other data in this paper do. There is a good antibody to mouse L1 ORF1 (validated by Alex Bortvin, Abcam AB216324), and it would be easy to perform and include those experiments on the specimens already in hand.

RESPONSE: We appreciate the reviewer's suggestion, and we agree that bulk RNA-seq does not provide single cell resolution. We only had a very limited amount of 3-month CNS tissue slides left in our freezer as all the rest of the tissues collected were utilized for the various experiments reported in the manuscript. We have attempted staining of these with the suggested antibody, but the degradation of the tissues was such that even our reliable GFP staining was imperfect, and we were not able to get any L1orf1p staining with the AB216324 antibody. In addition, that antibody was validated in gonadal tissues, not CNS. We hope the reviewer will accept that these are difficult and long-term experiments to do, and hope that the reviewer will accept that it was not possible for us to address this issue in the context of this revision. Certainly it's a good experiment to add to our next manuscript on this topic.

Minor issues

1) The authors keep referring to "clusters", defined as "large, spatially defined groups of L1-EGFP-labeled cells". The manner in which they are scored is described verbally (which is clear), but it would be very helpful to have some visuals on this. For example, in some of the images presented, draw a dashed line around what is being referred to as a "cluster".

RESPONSE: We appreciate the comment for clarification of visual representation. However, the designation of a "cluster" is a statistical measure across the entire motor cortex across 14 sections, and therefore it is not viable to draw dashed lines around each determined cluster in each figure. Figure 4, for example, is only a single section from a small field of view within the motor cortex. Including dashed lines we feel would be more confusing as they would not properly represent the actual measured clusters. The figures are for visual reference of the cluster identities.

2) Row designations in Fig. 2a, b are missing.

RESPONSE: This has been corrected and heat map row designators have been added.

3) Is the lifespan of mutant-TDP Tg mice known? They are missing from the 17 month timepoint.

RESPONSE: Although some hTDP-43-Q331K animals can live up to 2 years the survival is already greatly reduced at 17 months and therefore it was impossible to test an entire cohort of these animals.

4) Fig. 3: not clear what is being plotted here. Each dot corresponds to one mouse, and the position on the Y axis is the average number of GFP positive cells in that mouse? How were the violin outlines computed?

RESPONSE: Each dot corresponds to one animal. We quantified GFP-positive cells across 14 sections and plotted the GFP-positive cells/ section on the Y-axis. The violin plot outline was computed using GraphPad Prism.

5) Sentence line 709-711, meaning is not clear. What is meant by any upregulated RTE? Both significantly and non-significantly upregulated RTEs? Was there no significant overlap between significantly upregulated in WT hTDP and significantly upregulated in Q331K hTDP?

RESPONSE: There were only 4 significantly upregulated RTEs in the 3mo hTDP-43-Q331K motor cortex as shown in Fig 2c. However, there were 908 significantly upregulated RTEs in the 15mo hTDP-43-WT motor cortex. Due to these differences we compared all upregulated RTEs in the 3mo hTDP43-Q331K and significantly upregulated RTEs in the 15mo hTDP-43-WT animals and found a significant overlap. We decided to use the significantly upregulated RTEs in the 15mo hTDP-43-WT animals as the final set of RTEs that are upregulated as a result of TDP-43-related neurodegeneration.

6) Sentence on line 713-715: "We then compared . . . " Where is the result of this comparison? Something seems to be missing.

RESPONSE: In this statement we refer to supplementary table 2d and have added that to the text.

7) The authors keep referring to "replication" of L1, as evidenced by the L1-EGFP reporter. I would prefer to use "retrotransposition" – I believe it is a more descriptive and accurate term.

- **We appreciate the clarification by the reviewer and we have replaced the term replication with the more appropriate term retrotransposition throughout the manuscript.**

Have all data underlying the figures and results presented in the manuscript been provided?
Large-scale datasets should be made available via a public repository as described in the *PLOS Genetics* data availability policy, and numerical data that underlies graphs or summary statistics should be provided in spreadsheet form as supporting information.

Reviewer #1: None

We are unsure what Reviewer #1 is referring to as "None" for the data underlying the figures being provided. We have included all data for all figures in the supplemental tables included with the submission.

Reviewer #2: Yes
Reviewer #3: Yes

PLOS authors have the option to publish the peer review history of their article (what does this mean?). If published, this will include your full peer review and any attached files.

If you choose “no”, your identity will remain anonymous but your review may still be made public.

Do you want your identity to be public for this peer review? For information about this choice, including consent withdrawal, please see our Privacy Policy.
Reviewer #1: No
Reviewer #2: No
Reviewer #3: No

[NOTE: If reviewer comments were submitted as an attachment file, they will be attached to this email and accessible via the submission site. Please log into your account, locate the manuscript record, and check for the action link "View Attachments". If this link does not appear, there are no attachment files.]
